# Simultaneous visualization of RNA transcripts and proteins in whole-mount mouse preimplantation embryos using single-molecule fluorescence *in situ* hybridization and immunofluorescence microscopy

**DOI:** 10.3389/fcell.2022.986261

**Published:** 2022-10-04

**Authors:** Rasmani Hazra, David L. Spector

**Affiliations:** Cold Spring Harbor Laboratory, Cold Spring Harbor, NY, United States

**Keywords:** lncRNA, mRNA, mouse embryo, whole-mount, single-molecule RNA FISH, immunofluorescence

## Abstract

Whole-mount single-molecule RNA fluorescence *in situ* hybridization (smRNA FISH) in combination with immunofluorescence (IF) offers great potential to study long non-coding RNAs (lncRNAs): their subcellular localization, their interactions with proteins, and their function. Here, we describe a step-by-step, optimized, and robust protocol that allows detection of multiple RNA transcripts and protein molecules in whole-mount preimplantation mouse embryos. Moreover, to simultaneously detect protein and enable RNA probe penetration for the combined IF/smRNA FISH technique, we performed IF before smRNA FISH. We removed the zona pellucida, used Triton X-100 to permeabilize the embryos, and did not use a proteinase digestion step so as to preserve the antigens. In addition, we modified the IF technique by using RNase-free reagents to prevent RNA degradation during the IF procedure. Using this modified sequential IF/smRNA FISH technique, we have simultaneously detected protein, lncRNA, and mRNA in whole-mount preimplantation embryos. This reliable and robust protocol will contribute to the developmental biology and RNA biology fields by providing information regarding 3D expression patterns of RNA transcripts and proteins, shedding light on their biological function.

## Introduction

RNA sequencing technologies have revealed that a large part of the mammalian genome encodes for long non-coding RNAs (lncRNAs), which are greater than 200 nucleotides in length (Wang and Chang, 2011; Derrien et al., 2012; Djebali et al., 2012; Rinn and Chang, 2012). They primarily interact with DNA, mRNA, and protein to regulate gene expression and are generally expressed in a cell- or tissue-specific manner (Mercer et al., 2009). The expression of lncRNAs within the tissue and their subcellular localization are essential determinants of their molecular function. RNA fluorescence *in situ* hybridization (FISH) is a valuable technique to directly detect RNA molecules in the cell through the hybridization of labeled nucleic acid probes that target RNA (Singer and Ward, 1982; Singer et al., 1986; Schurter et al., 2002). However, standard RNA FISH using a nick translated probe is not sensitive enough to visualize moderate to low abundant lncRNAs. In contrast, single-molecule RNA fluorescence *in situ* hybridization (smRNA FISH), which is based on pools of short, labeled DNA oligonucleotides (Femino et al., 1998; Raj et al., 2008), can detect individual RNA molecules *in situ*, thereby affording it the sensitivity to detect even low abundance lncRNAs (Bergmann et al., 2015; Tripathi et al., 2015; Mehta-Mujoo et al., 2019; Chang et al., 2020). Moreover, this technique can allow for the quantification of RNA transcripts within the cell.

The recently developed, commercially available RNAscope technology can detect RNA *in situ* using fluorescent signal amplification upon target probe hybridization (Wang et al., 2012). RNAscope has been applied successfully to detect lncRNAs in sectioned tissues or cultured cells (Tripathi et al., 2018; Mehta-Mujoo et al., 2019; Hazra et al., 2021) and in intact (whole-mount) pre- and post-implantation mouse embryos (Hazra et al., 2021). The whole-mount method maintains tissue structural integrity and detection of lncRNA targets through smRNA FISH, which can be used to explore the spatiotemporal expression of specific genes of interest throughout the entire embryo. Although smRNA FISH can detect and quantify lncRNA transcripts, it cannot provide information about lncRNA-protein associations. However, by applying the sequential method of smRNA FISH and standard IF, one can detect both lncRNA and protein in the same sample (Tripathi et al., 2010). Though the concept is simple, and there are some existing protocols available for cells (Trotter et al., 2021) and tissue sections (Meng et al., 2018), to our knowledge, no such protocol has been made available for whole-mount mouse preimplantation embryos. The inner cell mass of the preimplantation embryo gives rise to the whole embryo, and trophectoderm gives rise to the placental tissues (Schrode et al., 2013). Thus, the preimplantation embryo is a model for studying *in vivo* cell fate specifications during mammalian development.

Combining IF with smRNA FISH methods in whole-mount preimplantation mouse embryos is difficult and may lead to artifacts and/or experimental failure. Here, we optimized RNase-free conditions for the standard IF method followed by the smRNA FISH method using an RNAscope probe set to ensure the successful, simultaneous detection of protein, lncRNA, and mRNA in whole-mount preimplantation mouse embryos. This smRNA FISH method provides exquisite cellular localization, which can be coupled with IF to identify specific cell populations and/or cells expressing certain proteins. This combined method is extremely valuable not only to detect lncRNAs but also to detect mRNAs and proteins in preimplantation mouse embryos: it can detect developmentally important genes and provide detailed quantitative information concerning expression patterns and expression levels within the developing embryo and tissue. It is a rapid and robust protocol that facilitates the simultaneous quantitative detection of multiple transcripts within an embryo. Importantly, in addition to revealing the spatial distribution of RNA molecules, the protocol allows the simultaneous determination of protein expression patterns and subcellular protein localization.

## Materials and equipment

### Mouse embryo collection and fixation

Pregnant mare’s serum gonadotropin (PMSG) (#HOR-272, ProSpec)
Human chorionic gonadotropin (hCG) (#C1063, Sigma Aldrich)
KSOM Mouse Embryo Media (#MR-121-D, Millipore Sigma)
EmbryoMax^®^ M2 Medium (#MR-015-D, Millipore Sigma)
Mineral oil (#10029, Ovoil, Vitrolife)
Acidic Tyrode’s solution (#MR-004-D, Sigma-Aldrich)
Paraformaldehyde (PFA) (#19200, Electron Microscopy Sciences)
Phosphate-buffered saline (PBS) (#14190-250, Life Technologies)
4-well chamber plate (#144444, Thermo Fisher)
Bovine serum albumin (BSA) (#15260037, Thermo Fisher)
PBA solution: PBS in 1% BSA.

### Immunofluorescence

Molecular Grade Water (RNase-, DNase-, Proteinase-free) (#786-73C, G-Biosciences)
Triton X-100 (#X100, Sigma-Aldrich)
PBX solution: 0.1% Triton X-100 in PBS.
Horse serum (#H1270, Sigma-Aldrich)
SUPERase•In RNase Inhibitor (#AM2696, Thermo Fisher)
Blocking solution: 2% horse serum in PBS and 10 μg/ml SUPERase•In RNase Inhibitor)
anti-Cdx2 (#MU392A-UC, BioGenex)
anti-Tead4 (#ab58310, Abcam)
Goat anti-mouse IgG (Alexa Fluor 488) (#ab150113, Abcam)
96-well round bottom cell culture plate (#07-200-95, Thermo Fisher)
Agarose (#A0701-25G, Millipore Sigma)
Glass bottom dishes (#P06G-1.5, MatTek)

### smRNA FISH

20x saline-sodium citrate (SSC) buffer (#R020, G- Biosciences)
SSCT (0.2 × SSC buffer with 0.01% Tween-20)
RNAscope Multiplex Fluorescent V2 Assay (#323120, Advanced Cell Diagnostics)
Advanced Cell Diagnostics (ACD) RNAscope 3-plex Positive Control Probe (#320881)
ACD RNAscope 3-plex Negative Control Probe (#320871)
Mouse Platr4 probe set (#300031, RNAscope Target Probe C1)
Mouse Malat1 probe set (#313391, RNAscope Target Probe C1)
Mouse Pou5f1 probe set (#501611, RNAscope Target Probe C2)
Opal 570 and Opal 690 (#FP1488001KT and # FP1497001KT, Akoya Biosciences)
4’, 6-diamidino-2-phenylindole (DAPI) (#D1306, Thermo Fisher)
RNase A (#EN0531, Thermo Fisher)

### Equipment

Water Jacketed CO_2_ incubator, 37°C, 5% CO_2_ with HEPA filtration (model 3120, Thermo Fisher)
Incubator (#I5110A, Stellar Scientific)
Microscope, inverted (model TS100 or equivalent, Nikon)
Stereoscope dissection microscope (model MZ12.5 or equivalent, Leica)
Microscope, confocal (LSM 780, Zeiss)
0.22 µM filter unit, such as Stericup-GV (#SCGVU05RE, Millipore)
Mouthpiece for embryo manipulation (#72150, Kitazato)
Micro-dissecting scissors (#RS-5610, Roboz)
Forceps (#RS-4984, Roboz)
Pipette set (cat. no. 1075-0810, USA Scientific)
Serological pipettes, 10 ml (cat. no. 1071-0810, USA Scientific)
Syringes, 5 ml (cat. no. 309646, BD) with 27G needle (cat. no. 305109, BD)
4-well tissue culture treated plate (cat. no. 353654, Corning Life Sciences)

## Methods

### Mouse embryo collection and fixation

Four-to five-week-old female mice were injected with 5 IU of PMSG; 48 h later, they were injected with 5 IU of hCG, then immediately placed in a cage with a fertile stud male for mating. On the morning of the next day (between 9 and 10 a.m.), the presence of vaginal plugs was checked. Mated female mice were placed in a new cage separate from the males; this time point was considered embryonic day (E) 0.5. Preimplantation embryos—blastocysts (E3.5) were collected 3 days later.

Before the embryos were collected from the uterus of the pregnant female mice on the desired day, M2 medium was warmed to 37°C. Then, 4% PFA in PBS, 1% agarose in saline, and thawed acidic Tyrode’s solution were prepared. A glass Pasteur pipette was pulled using an open flame to draw a capillary end at the tip to manipulate the embryos. Pregnant female mice were sacrificed by CO_2_ inhalation and cervical dislocation. Preimplantation mouse embryos (blastocysts) were collected by flushing the uterus with an M2 medium. The embryos were collected using the mouth-controlled, glass Pasteur pipette with a capillary end, transferred to a new dish, and further washed by moving them through two to three drops of fresh M2 media. A stereoscope dissection microscope was used to locate the embryos. The zona pellucida was removed from the blastocysts by briefly washing it in acidic Tyrode’s solution; as soon as the zona pellucida was dissolved, all embryos were transferred back to the M2 medium. Notably, the embryos were very sticky after removing the zona pellucida. The glass pipette was coated with 1% BSA before use to both reduce the adherence of the embryos to the glass surface and reduce the risk of damage to or loss of the embryos.

Next, 500 µL of PBA was added to one of the wells of the coated 4-well tissue culture dish, and 500 µL of 4% PFA was added to another well. The embryos were briefly washed with PBA before fixing them in 4% PFA for 20 min at room temperature (RT), then washed three times in PBX for 5 min. The fixed embryos were used to perform the IF followed by smRNA FISH assay.

Note: At this stage, the fixed embryos can be stored for up to 2 days at 4°C before proceeding to the next step. To store the embryos, cover the PBS containing the embryos with a layer of mineral oil to prevent evaporation.

### Immunofluorescence

Standard IF was performed (Saiz et al., 2016) with minor modifications. The desired number (e.g., 20 embryos) of fixed embryos were transferred to a coated (1% agarose) round bottom 96-well plate to perform all the steps involved in IF and smRNA FISH. Notably, the round bottom 96-well plate was coated with 1% agarose to prevent the embryos from sticking to the plastic, and the 96-well plate minimizes the use of reagents. The embryos were incubated in 1% Triton X-100 in PBS for 30 min on ice for permeabilization. Then, the embryos were washed three times in PBX for 5 min each at RT, blocked in blocking solution for 1 h at RT, and incubated in primary antibody diluted in blocking solution (1:200 for anti-Cdx2 and 1:100 for anti-Tead4) overnight at 4°C. The solution was covered with a layer of mineral oil to prevent evaporation. The next day, the embryos were washed in PBX three times for 5 min each at RT, then incubated in secondary antibody diluted in blocking solution (1:500, Alexa Fluor 488-Goat anti-mouse IgG) for 2 h at 4°C. The embryos were washed in PBX three times for 5 min each at RT and followed up with the smRNA FISH procedure.

### smRNA FISH

smRNA FISH was performed using ACD RNAscope technology according to the manufacturer’s instructions, with modifications. smRNA FISH method was initiated by an additional fixation step using 4% PFA for 10 min at RT. Then, the embryos were rinsed three times in fresh PBX solution (0.1% Triton X-100 in PBS). Please note that the protease digestion step was skipped. The embryos were then incubated for 2 h at 40 ± 2°C in a probe-containing solution (RNAscope Positive Control Probe, RNAscope Negative Control Probe, lncRNAs *Platr4* and *Malat1* and mRNA *Pou5f1* probes. PBS was used as a no probe control. Note: We recommend using (RNase-DNase-, Proteinase-free) molecular grade water and Ca_2_^+^/Mg_2_^+^-free PBS to dilute any buffers, which preserves both FISH and IF signals.

Due to the specific gravity of the probe-containing solution, embryos tend to float; they also become transparent, which makes it challenging to find them in the solution. Using the stereoscope dissection microscope, it is crucial that the embryos are at the bottom of the well, so they are fully submerged during the 2-h incubation process. After the probe hybridization, the embryos were washed three times for 5 min each in SSCT at RT. We did not use ACD wash buffer because it disintegrates the embryos. After washing the embryos, we performed a series of incubations with pre-amplifier and amplifier reagents (Amp 1, Amp 2, and Amp 3) and fluorophore at 40 ± 2°C for 15–30 min each, according to manufacturers’ protocols. After every incubation, the embryos were washed twice in SSCT for 5 min at RT. After the final wash, the embryos were stained with DAPI solution and left overnight at 4°C, and the solution was covered with a layer of mineral oil. The next day, the embryos were washed twice in PBS for 5 min at RT and then transferred to a MatTek dish containing a drop of PBS for imaging.

### Confocal imaging

Whole-mount preimplantation mouse embryos were imaged using a Zeiss LSM 780 laser-scanning confocal microscope equipped with a GaAsP detector with laser lines at 405, 488, 561, and 633 nm. *Z*-stack images were acquired with a 63x/1.4 oil immersion objective lens and collected in 12-bit and the pixel size in the software was set to zoom 1.5 of 88 nm in x, y with a z-spacing of 1.0 µm with Zen 2011 SP7 software. Line averaging of two–four and pinhole size was set to one to 1.68 Airy Unit (AU) were used to balance a good SNR, optical sectioning resolution and time to collect 3D datasets, the GaAsP detectors gain were set to achieve strong signal without saturating, the digital offset/background was set to avoid any pixels reading zero intensity values. ZEN Microscopy software was used for image processing and analysis.

## Results

### A protocol for preimplantation mouse embryo collection, fixation, IF, and smRNA FISH

**FIGURE 1:**
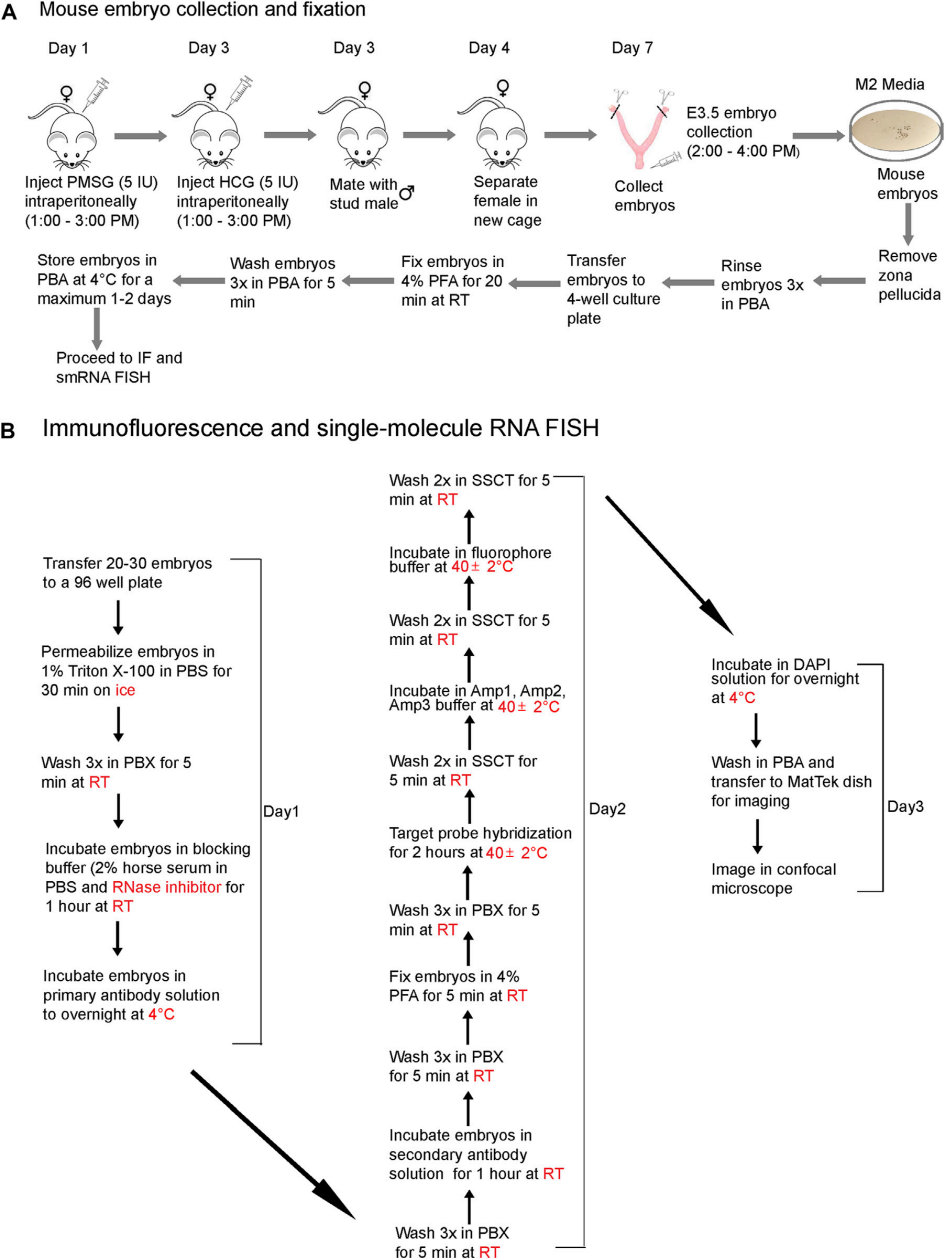
A workflow diagram for **(A)** mouse embryo collection and fixation and **(B)** the IF and smRNA FISH protocol.

We present a step-by-step protocol to superovulate mice, collect preimplantation embryos, and fix them in PFA (Figure 1A), followed by optimized, combined IF and smRNA FISH techniques (Figure 1B) to detect proteins and lncRNAs in intact preimplantation mouse embryos. The workflow diagram in Figure 1 outlines all the steps in this protocol. We have also provided images of live blastocysts (Supplementary Figure S1)

### Combining smRNA FISH and IF to detect lncRNA and proteins in preimplantation embryos

**FIGURE 2:**
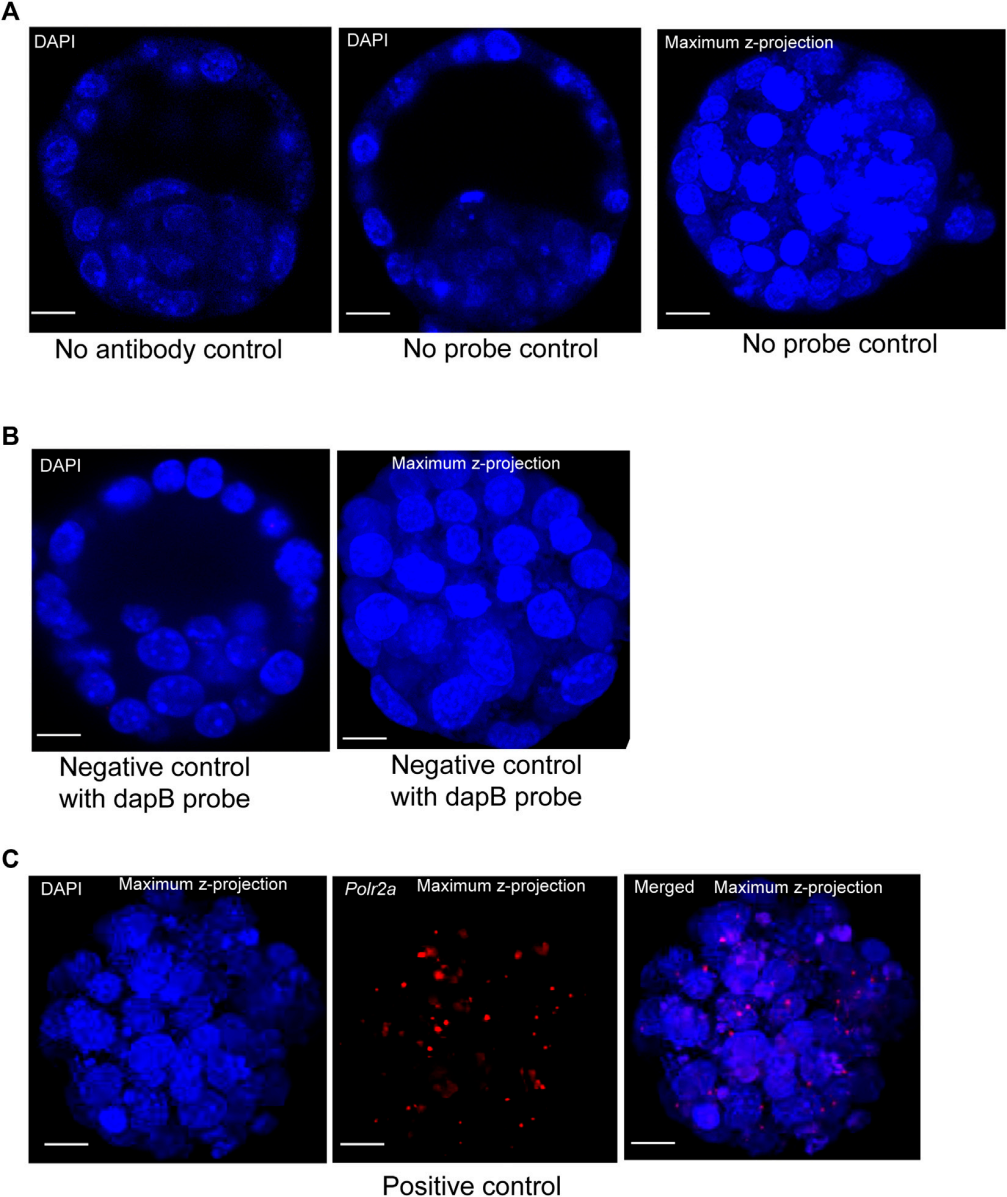
**(A)** Image represents no primary antibody control. DAPI stain to the nucleus. Image is shown at a single z-plane, scale bars: 12 μm. Images are shown of a no smRNA FISH probe control. DAPI stain to the nucleus. Images are shown at a single z-plane and maximum intensity z-projection, scale bars: 12 μm. **(B)** A negative-control probe targeting the *E. coli* dapB gene. DAPI stain to the nucleus. All the images are shown at a single z-plane and maximum intensity z-projection, scale bars: 12 μm. **(C)** Images are shown of a positive-control probe targeting the mouse *Polr2a* gene. The maximum intensity Z-projection of mouse blastocysts images are shown here, with scale bars: 12 μm.

To visualize protein and lncRNA in the same sample, we first optimized the hybridization conditions using no antibody control, no probe control (Figure 2A), and negative and positive control probes in mouse blastocysts (Figures 2B,C). We have used the ACD RNAscope proprietary probes for the Positive and Negative control samples. Each of these probes was designed based on NCBI sequence data. Probes consist of 20 oligonucleotide pairs and each pair has a double Z configuration: One side of the Z is complementary to the specific RNA transcript and the other side is complementary to the amplifier DNA sequence (Wang et al., 2012)

The *Escherichia coli* dihydrodipicolinate reductase (dapB) gene mRNA was used as the negative control to verify the non-specific signal, and our data showed that we achieved complete negative staining in our sample (Figure 2B). We have detected the positive control probe for the housekeeping gene *RNA polymerase 2a* (*polr2a*) mRNA (detection channel-1, C1) as red punctuate dots (Figure 2C). The expression level of *Polr2a* mRNA was low in the cells, which others have shown in mouse preimplantation embryos, cultured mammalian cells, and tissue (Wang et al., 2012; Xie et al., 2018).

To detect the localization of a lncRNA and protein in the same sample, we first performed smRNA FISH using an ACD RNAscope probe and reagents, followed by standard IF (smRNA FISH - IF). The sequential smRNA FISH and standard IF can be technically challenging because of RNA degradation. However, previously this approach has been used to detect RNA and protein in nonadherent mammalian cells (Arrigucci et al., 2017), cultured mouse neurons (Eliscovich et al., 2017), paraffin-embedded tissue sections (Kwon et al., 2020), zebrafish (Gross-Thebing et al., 2014) and the *C. elegans* germline (Yoon et al., 2016). Therefore, we performed sequential smRNA FISH and IF in preimplantation mouse embryos. We used an anti-Cdx2 antibody to detect Cdx2 protein in mouse blastocysts. The transcription factor Cdx2 is expressed in the TE lineage and is vital for TE differentiation (Meissner and Jaenisch, 2006). We also used a target probe set for the lncRNA *Platr4*, which is crucial for differentiation and expressed in both the TE and inner cell mass (Hazra et al., 2021). However, using this sequential combination of the two techniques, smRNA FISH followed by IF, we were unable to detect the lncRNA (*Platr4*) signals but did detect the protein (Cdx2) signal. We reasoned that this failure to detect the lncRNA signal could be due to 1) reagents (e.g., blocking buffer, crude antibody solution): used in IF were not RNase-free, 2) the order of performing IF and smRNA FISH because in cell culture systems when using these two methods simultaneously in some cases the procedures work when IF is done first and in other cases (different targets) it works when the RNA FISH is done first.

### Simultaneous detection of proteins and lncRNAs in whole-mount preimplantation mouse embryos

**FIGURE 3:**
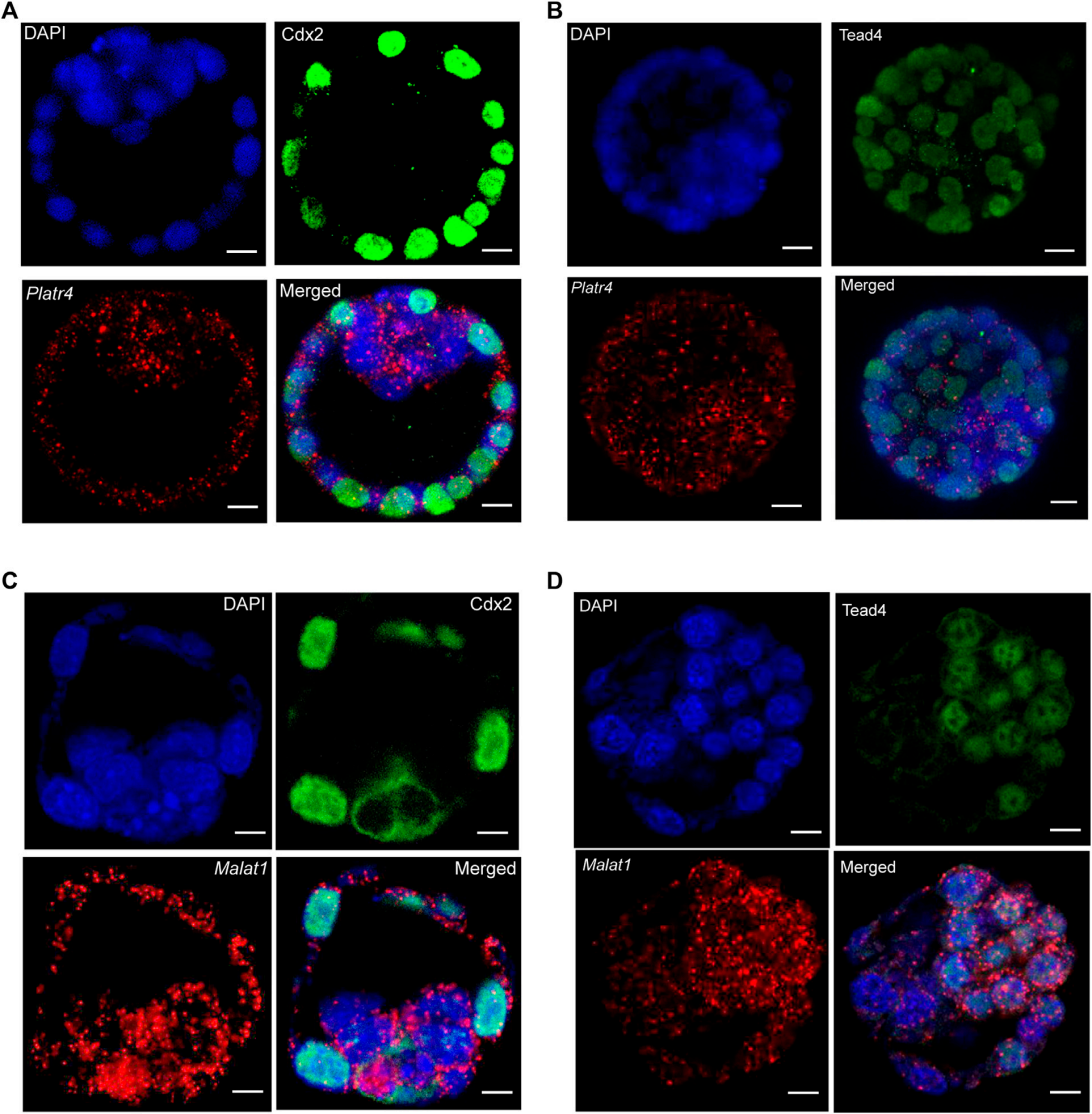
**(A)** Combining IF and smRNA FISH using a *Platr4* lncRNA probe and anti-Cdx2 antibody **(B)** Combining IF and smRNA FISH using a *Platr4* lncRNA probe and anti-Tead4 antibody. **(C)** Combining IF and smRNA FISH using a *Malat1* lncRNA probe and anti-Cdx2 antibody. **(D)** Combining IF and smRNA FISH using a *Malat1* lncRNA probe and anti-Tead4 antibody. Nuclei were counterstained with DAPI. Mouse anti-Cdx2 and mouse anti-Tead4 primary and Alexa Fluor 488-conjugated goat anti-mouse secondary antibodies were used for IF detection. All the images are shown at a single z-plane, scale bars, 12 μm.

Next, we optimized a protocol where an RNase-free IF method was performed first, followed by smRNA FISH in whole-mount mouse blastocysts. We further modified the smRNA FISH method in four critical ways: 1) we used RNase-free reagents to perform IF, 2) we added an additional post-fixation step after incubation with the secondary antibody but before starting the probe hybridization for smRNA FISH to prevent the removal or displacement of the antibody, 3) we eliminated the protease digestion treatment step to prevent loss of protein signal. Interestingly, these optimized modifications allowed us to successfully detect both IF signals for the Cdx2 and Tead4 proteins and the smRNA FISH signals for the *Platr4* and *Malat1* lncRNAs (Figures 3A–D, Supplementary Figures S2A, B). In the preimplantation mouse embryo, Tead4 is required to establish the TE-specific transcriptional program and segregate the TE lineages from the ICM (Home et al., 2012). In addition, Tead4 is essential for the expression of TE-specific transcription factor Cdx2 (Home et al., 2012). Our results also have shown the TE-specific expression of the Cdx2 protein (Figures 3A,C), whereas Tead4 expression is present in both TE and ICM lineages (Figures 3B,D). We have also found that both lncRNAs *Platr4* and *Malat1* are expressed in the TE and ICM lineages (Figures 3A–D, Supplementary Figures S2A, B).

### Detection of multiple RNA transcripts in combination with IF in whole-mount mouse preimplantation embryos

**FIGURE 4:**
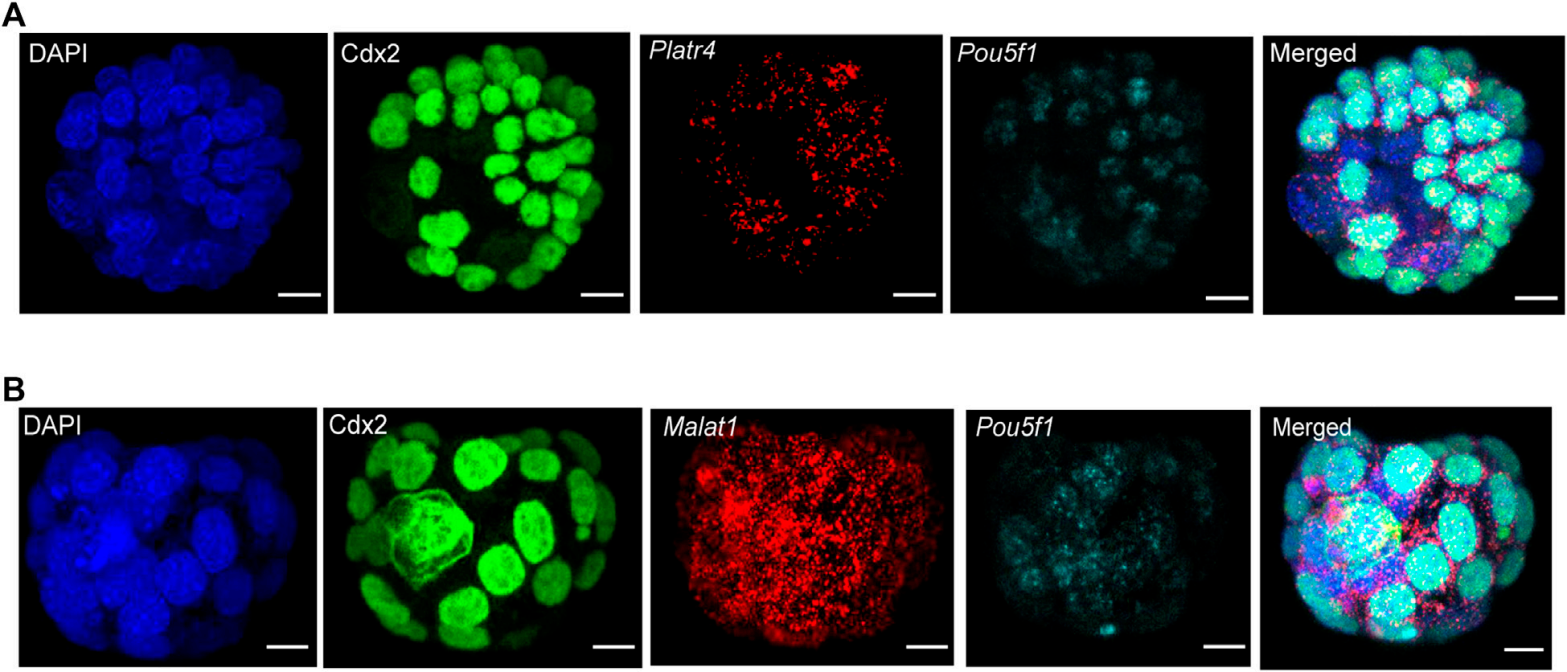
**(A)** Detection of multiple RNA transcripts, such as lncRNA *Platr4*, mRNA *Pou5f1,* and Cdx2 protein in 3D-mouse blastocysts. Nuclei were counterstained with DAPI. The green color represents the Cdx2 protein signal, and red and cyan punctate dots represent the RNA signal for *Platr4* and *Pou5f1* transcripts. All images are shown at the maximum intensity, the Z-projection, scale bars: 12 μm. **(B)** Detection of *Malat1* lncRNA, *Pou5f1* mRNA, and Cdx2 protein in 3D-mouse blastocysts. Nuclei were counterstained with DAPI. The green color represents the Cdx2 protein signal, and red and cyan punctate dots represent the RNA signal for *Malat1* and *Pou5f1* transcripts. All images are shown at the maximum intensity, the Z-projection, scale bars: 12 μm.

We further sought to detect multiple RNA transcripts simultaneously in the three-dimensional context of preimplantation mouse embryos. Therefore, we have used two different RNA transcripts (lncRNA and mRNA) smRNA FISH probes in combination with either Tead4 or Cdx2 protein (Figures 4A,B). For the visualization of multiple RNA transcripts, we have shown expression of either *Platr4* or *Malat1* lncRNAs and *Pou5f1* mRNA, which are expressed in the ICM of mouse blastocysts (Ohta et al., 2008). In addition, we showed detection of two mRNA transcripts (*Pou5f1* and *Polr2a*) in combination with Tead4 protein (Supplementary Figure S3). Thus, our combined methods successfully detected protein, lncRNA, and mRNA in the same sample, indicating that this method is robust and suitable for visualizing multiple biological molecules in the three-dimensional context of developing mouse embryos.

## Discussion

The coupling of RNA FISH and IF labeling in a whole-mount sample represents a powerful tool to visualize spatial associations between RNA transcripts and proteins in the cells/embryos while preserving the intact cell or tissue structure (Prasanth et al., 2005). For example, by combining IF and RNA FISH in human female IMR90 (normal diploid) interphase cells, Boggs et al. could detect H3 methylation at Lys9 colocalizing with the XIST RNA domain (Boggs et al., 2002). Furthermore, an advantage of this combined protocol is that it can simultaneously detect various RNAs, proteins, and epigenetic modifications (Ogawa and Ogawa, 2021). These combined protocols have also been applied to cultured mammalian cells (Arrigucci et al., 2017; Eliscovich et al., 2017), and in other biological systems, such as *Caenorhabditis elegans*, zebrafish, and Drosophila, either in sections or whole-mount (Toledano et al., 2012; Yoon et al., 2016; Collins and Dunleavy, 2018; He et al., 2020). Recently, such an approach has been used to detect retrotransposons in preimplantation mouse embryos using an smRNA FISH protocol with Stellaris probes (Percharde et al., 2021). These studies were performed using RNA FISH for protein-coding transcripts and IF for protein. Here, we show for the first time that smRNA FISH and IF can be combined to detect lncRNAs and proteins in whole-mount mouse preimplantation embryos.

In contrast to a protein-coding transcript, the majority of lncRNAs are generally expressed at a lower level (Mercer et al., 2011), which makes many lncRNAs challenging to visualize using the standard smRNA FISH method, which does not use a double ‘Z probe’ configuration and amplification of transcript-specific probes (Okamoto et al., 2004; Okamoto, 2018; Percharde et al., 2021). Interestingly, the commercially available RNAscope technology, with its unique single-stranded DNA ‘Z-probe’ pairs, signal amplification strategy, and labeling probes by branched DNA-amplification trees, enables one to visualize of transcript-specific signals with a high signal-to-noise ratio (Wang et al., 2012; Xie et al., 2018). Using this RNAscope technology with some modifications, we successfully detected both the low and high abundant lncRNAs *Platr4* and *Malat1* in whole-mount preimplantation embryos (Figure 3). Furthermore, using this technology, we have efficiently identified multiple RNA transcripts simultaneously with the combination of proteins in whole-mount mouse preimplantation embryos (Figure 4 and Supplementary Figure S3). Thus, we demonstrate a combined approach of smRNA FISH with IF in whole-mount preimplantation embryos to detect lncRNA, mRNA, and protein.

Additionally, whole-mount embryo staining is advantageous compared to staining sections because it preserves the embryo shape and structure and retains the cells’ accurate localization. Here, we present an optimized protocol that simultaneously detects protein, lncRNA, and mRNA in whole-mount preimplantation mouse embryos. Of particular interest, our IF/smRNA FISH protocol can detect both low and high abundant lncRNAs in whole-mount mouse embryos. Thus, this method will be useful to both the lncRNA and developmental biology fields, as it is fast, reliable, robust, and utilizes readily available reagents. In conclusion, this versatile combined IF and smRNA FISH method can easily be incorporated into the toolbox of any laboratory to address scientific questions related to lncRNAs that cannot be readily answered using other approaches.

## Data Availability

The raw data supporting the conclusion of this article will be made available by the authors, without undue reservation.

## Supplementary material

Supplementary Figure S1 Bright Field images of live mouse blastocysts before fixing in PFA. Scale bar: 100 μm.

Supplementary Figure S2
**(A)** Combining IF and smRNA FISH using a Malat1 lncRNA probe and anti-Cdx2 antibody **(B)** Combining IF and smRNA FISH using a Platr4 lncRNA probe and anti-Tead4 antibody. Nuclei were counterstained with DAPI. Mouse anti-Cdx2 and mouse anti-Tead4 primary and Alexa Fluor 488-conjugated goat anti-mouse secondary antibodies were used for IF detection. The green color represents either Cdx2 or Tead4 IF signals, and red punctate dots for smRNA FISH signals of Platr4 or Malat1. All images are shown as maximum intensity Z- projection, scale bar: 12 μm.

Supplementary Figure S3 Visualization of Polr2a and Pou5f1 mRNAs in combination with Tead4 IF signal. Mouse anti-Tead4 primary and Alexa Fluor 488-conjugated goat anti-mouse secondary antibodies were used for IF detection. The green color represents Tead4 IF signals, and red and cyan punctate dots for smRNA FISH signals of Polr2a and Pou5f1. All images are shown as maximum intensity Z-projection, scale bar: 12 μm.
