# Supplementary figures and images for: Simultaneous visualization of RNA transcripts and proteins in whole-mount mouse preimplantation embryos using single-molecule fluorescence in situ hybridization and immunofluorescence microscopy
Source: Front Cell Dev Biol. 2022 Oct 4;10:986261. doi: 10.3389/fcell.2022.986261 (PMC9577017; doi:10.3389/fcell.2022.986261)

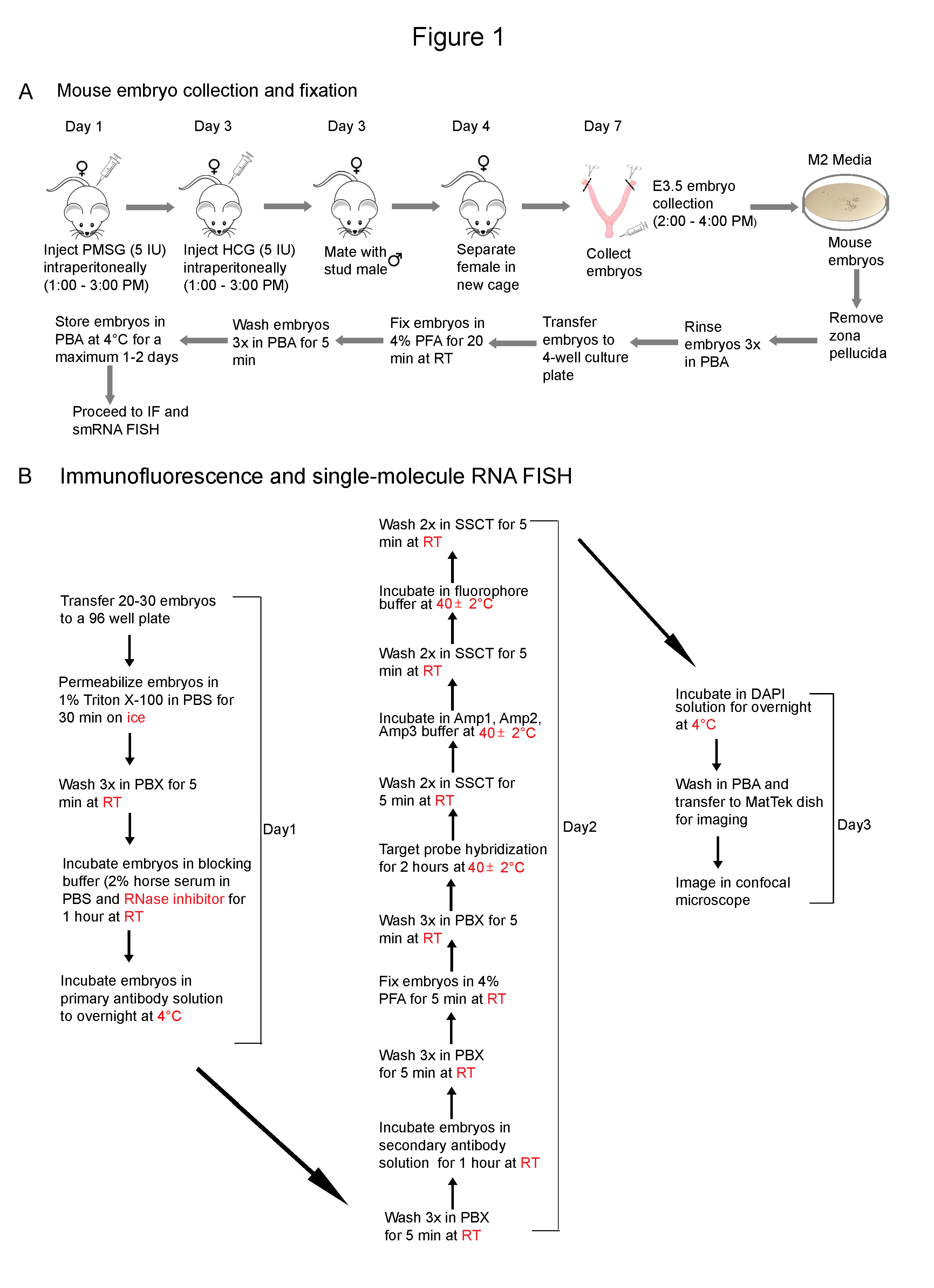

Supplement: Supplementary file 1 [file Figure1.TIFF]

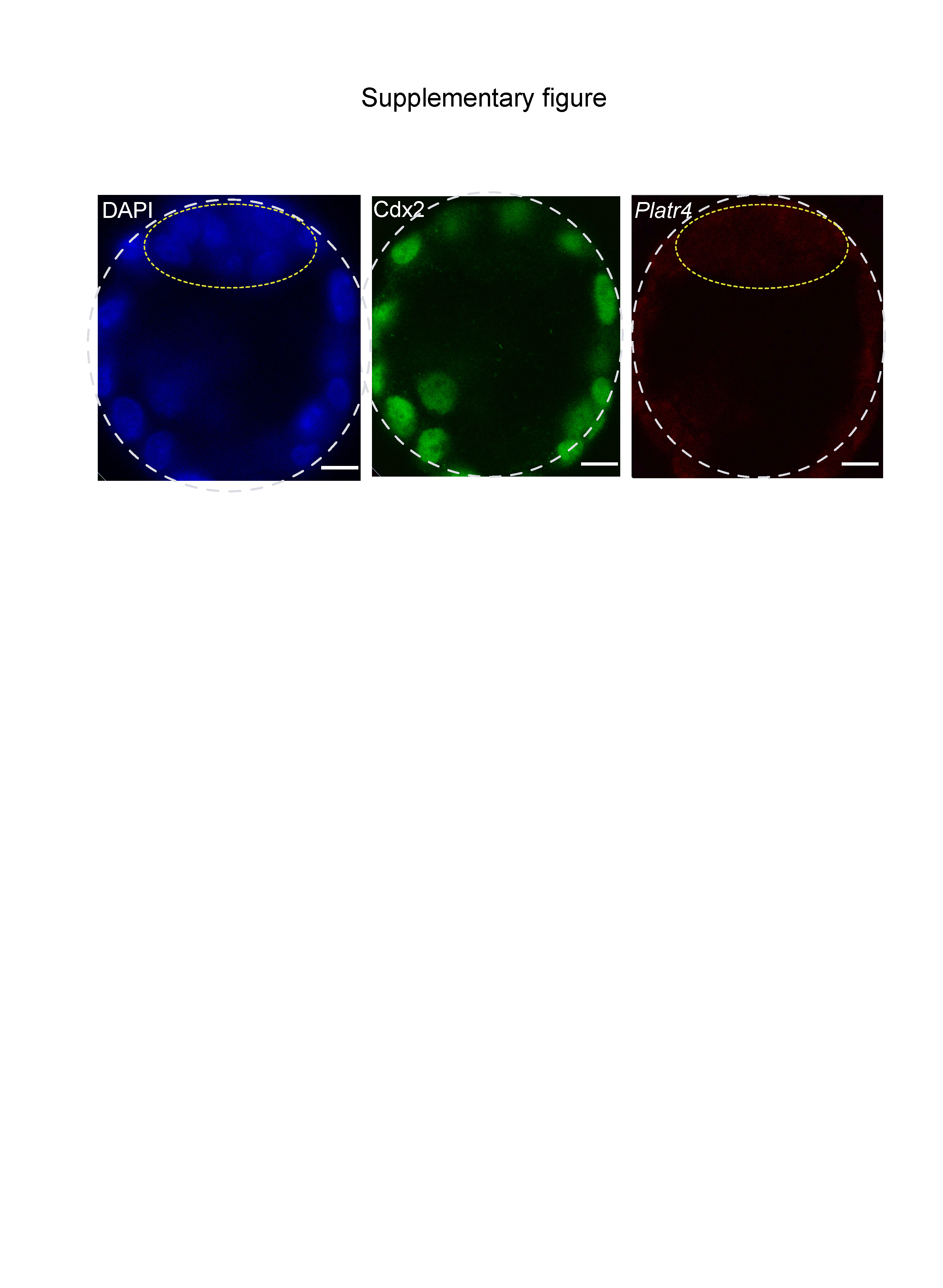

Supplement: Supplementary file 3 [file Figure2.TIFF]

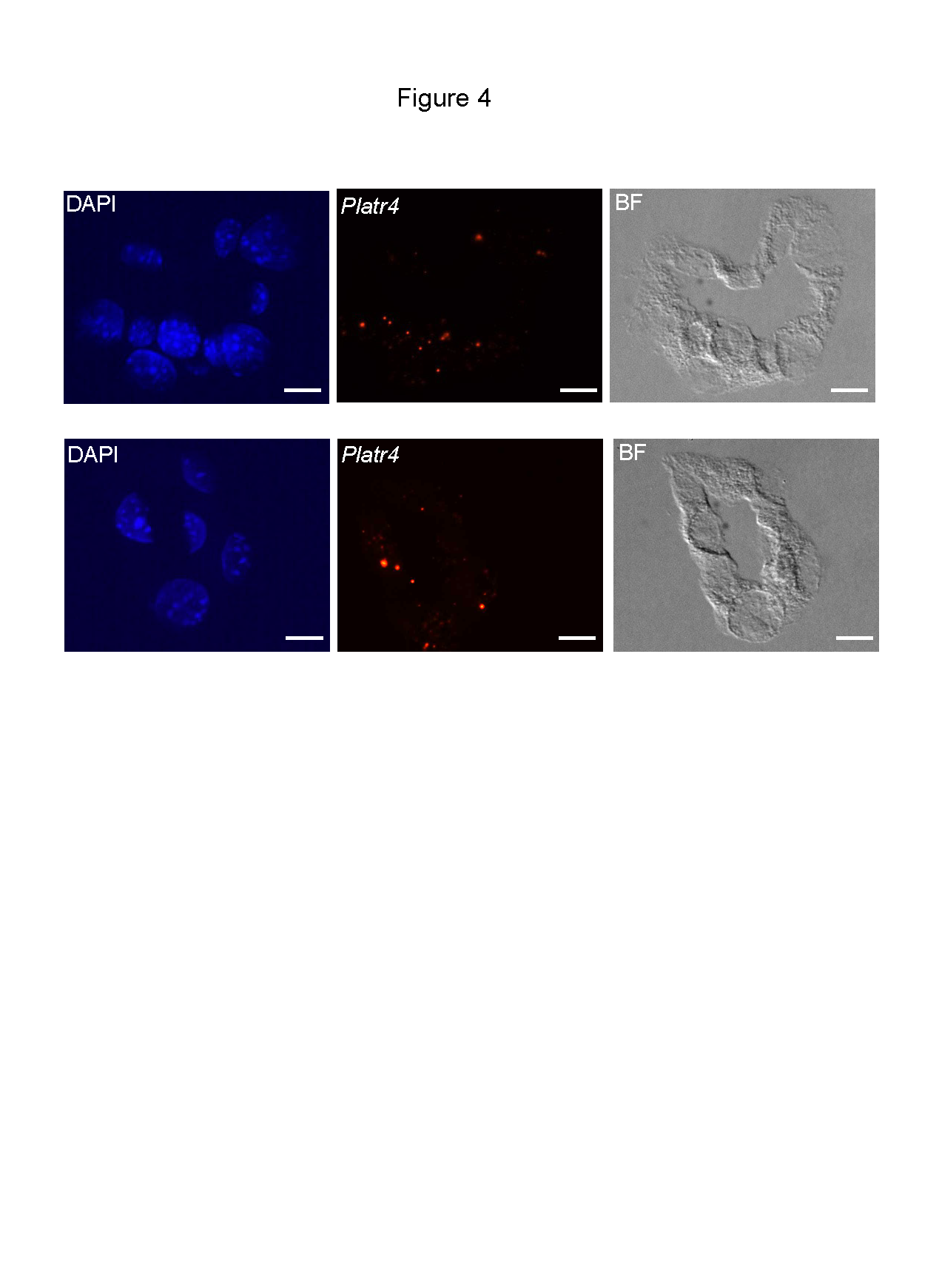

Supplement: Supplementary file 4 [file Figure3.TIFF]
