# Supplementary material for: Simultaneous visualization of RNA transcripts and proteins in whole-mount mouse preimplantation embryos using single-molecule fluorescence in situ hybridization and immunofluorescence microscopy
Source: Front Cell Dev Biol. 2022 Oct 4;10:986261. doi: 10.3389/fcell.2022.986261 (PMC9577017; doi:10.3389/fcell.2022.986261)
Supplement: Supplementary file 2 [file DataSheet1.pdf]

Supplementary Figure 1

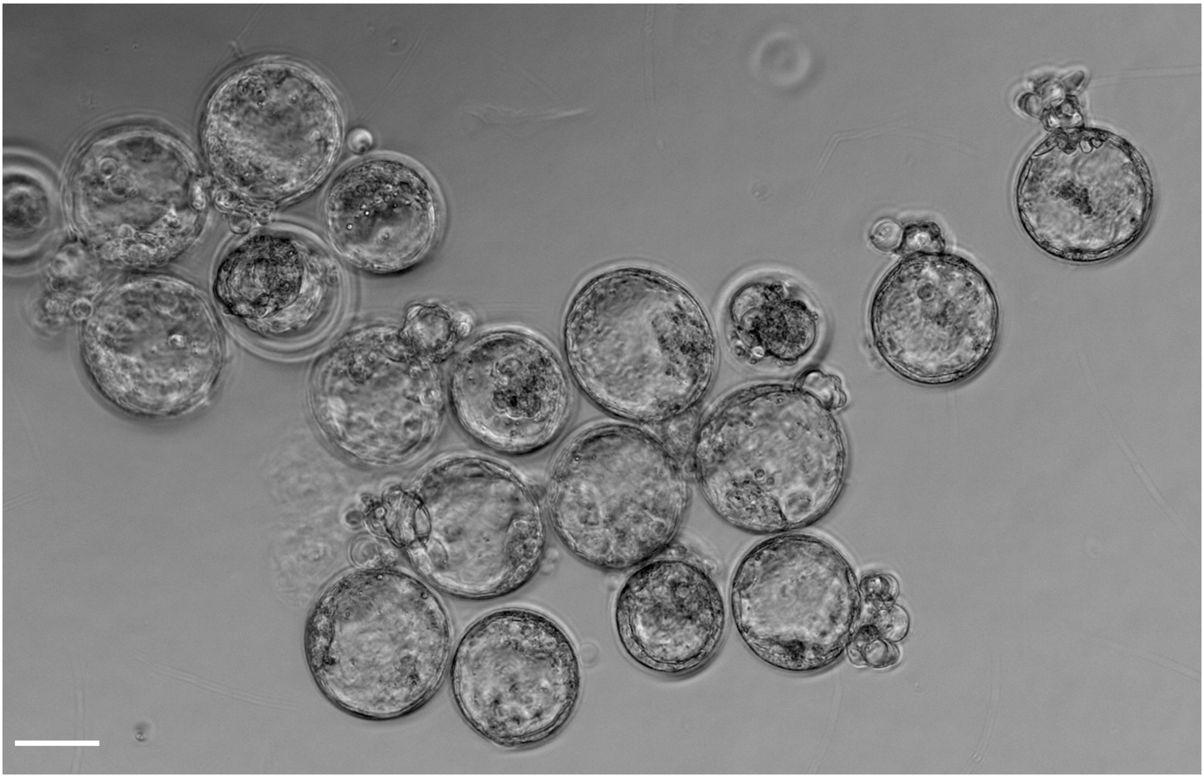

Supplementary Figure 2

A

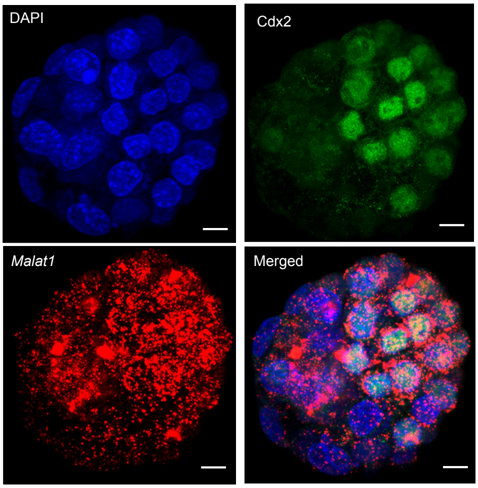

Maximum z-projection

B

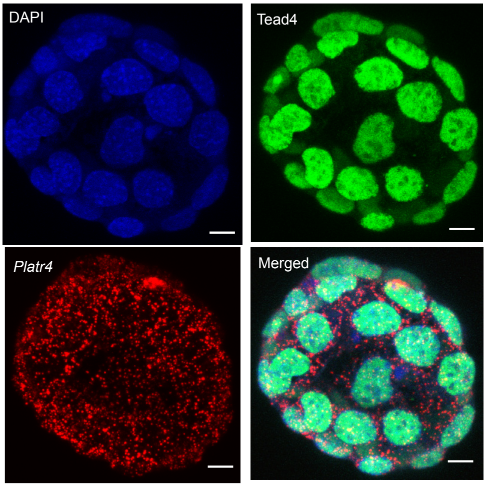

Maximum z-projection

Supplementary Figure 3

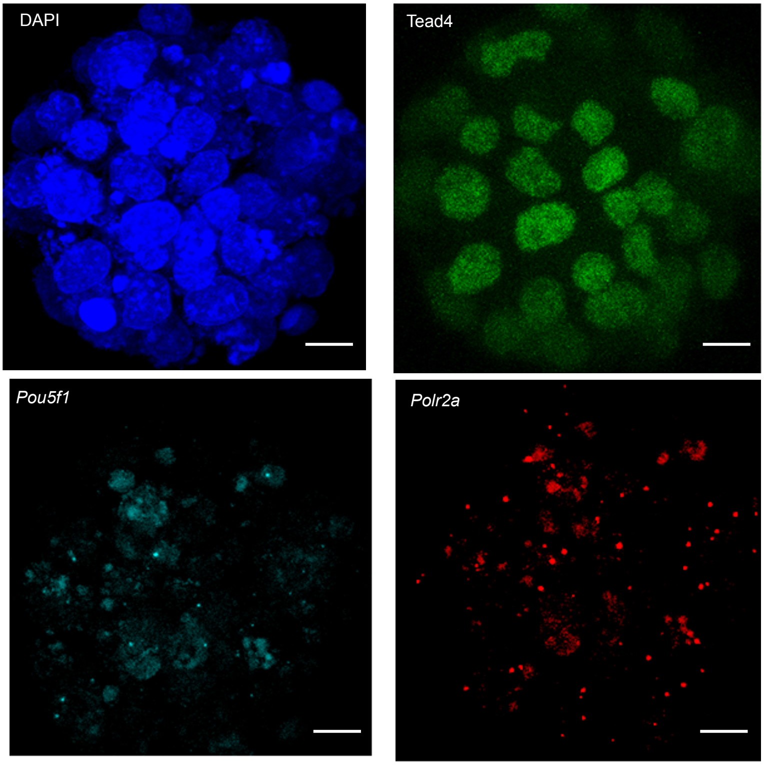

Maximum z-projection

Supplementary Figure Legends

Supplementary Figure 1: Bright Field images of live mouse blastocysts before fixing in PFA. Scale bar: 100 µm

Supplementary Figure 2: (A) Combining IF and smRNA FISH using a *Malat1* lncRNA probe and anti-Cdx2 antibody (B) Combining IF and smRNA FISH using a *Platr4* lncRNA probe and anti-Tead4 antibody. Nuclei were counterstained with DAPI. Mouse anti-Cdx2 and mouse anti-Tead4 primary and Alexa Fluor 488-conjugated goat anti-mouse secondary antibodies were used for IF detection. The green color represents either Cdx2 or Tead4 IF signals, and red punctate dots for smRNA FISH signals of *Platr4* or *Malat1*. All images are shown as maximum intensity Z-projection, scale bar: 12 µm.

Supplementary Figure 3: Visualization of *Polr2a* and *Pou5f1* mRNAs in combination with Tead4 IF signal. Mouse anti-Tead4 primary and Alexa Fluor 488-conjugated goat anti-mouse secondary antibodies were used for IF detection. The green color represents Tead4 IF signals, and red and cyan punctate dots for smRNA FISH signals of *Polr2a* and *Pou5f1*. All images are shown as maximum intensity Z-projection, scale bar: 12 µm.
